# Distinct Pharmacodynamic Complement Biomarker Signatures Define Iptacopan and Pegcetacoplan Treatment in C3 Glomerulopathy

**DOI:** 10.1016/j.ekir.2026.107087

**Published:** 2026-09-18

**Authors:** Yuzhou Zhang, Héctor Martín Merinero, Anna R. Carmen, Kameron Kruger, Renee X. Goodfellow, Dingwu Shao, Sarah M. Roberts, Angela F.M. Nelson, Nicole C. Meyer Horning, Sydney S. Jellison, Didier Mandelbrot, Neetika Garg, Carla M. Nester, Richard J.H. Smith

**Affiliations:** 1 Molecular Otolaryngology and Renal Research Laboratories, Carver College of Medicine, University of Iowa, Iowa City, Iowa, USA; 2 Division of Nephrology, Department of Medicine, University of Wisconsin School of Medicine and Public Health, Madison, Wisconsin, USA

**Keywords:** alternative pathway, C3 glomerulopathy, complement biomarkers, iptacopan, nephritic factors, pegcetacoplan, pharmacodynamics

## Abstract

**Introduction:**

C3 glomerulopathy (C3G) is driven by alternative pathway (AP) dysregulation. Iptacopan inhibits factor B, whereas pegcetacoplan inhibits C3/C3b, suggesting distinct pharmacodynamic (PD) biomarker profiles.

**Methods:**

We conducted an exploratory, nonrandomized observational cohort study of patients with biopsy-confirmed C3G treated with iptacopan (*n* = 9) or pegcetacoplan (*n* = 9), using paired pretreatment and approximately 3-month posttreatment samples. Complement activity, protein levels, activation products, autoantibodies, and genetic findings were assessed. Profiles were classified as PD-concordant (PDC), largely PD-concordant (LPDC), or PD-discordant (PDD) using treatment-specific signatures.

**Results:**

Both treatments suppressed AP activity and terminal pathway activation but produced distinct profiles. The expected PDC signature for iptacopan included increases in C3, factor B, C5, properdin, and CH50 and reductions in complement activation products (C3a, C3c, C3d, Bb, and sC5b-9). The expected pegcetacoplan signature included supranormal increases in C3, C3c, and properdin, reductions in C3a, C3d, Bb, and sC5b-9, and generally preserved or increased CH50. PDC profiles occurred in 6 of 9 patients in each cohort. LPDC profiles occurred in 3 iptacopan-treated patients, all nephritic factor-positive, and 2 pegcetacoplan-treated patients with nephritic factors or a likely pathogenic *C3* variant. One patient with severe factor H deficiency showed a PDD profile during pegcetacoplan treatment and again in a descriptive posttransplant sample during subsequent iptacopan treatment.

**Conclusion:**

Iptacopan and pegcetacoplan produce distinct, mechanism-specific PD signatures in C3G. Pathway-based biomarker assessment may distinguish expected treatment effects from incomplete or discordant biochemical control. These categories describe PD patterns rather than clinical efficacy and require validation against clinical outcomes.

## Graphical abstract

**Figure:**
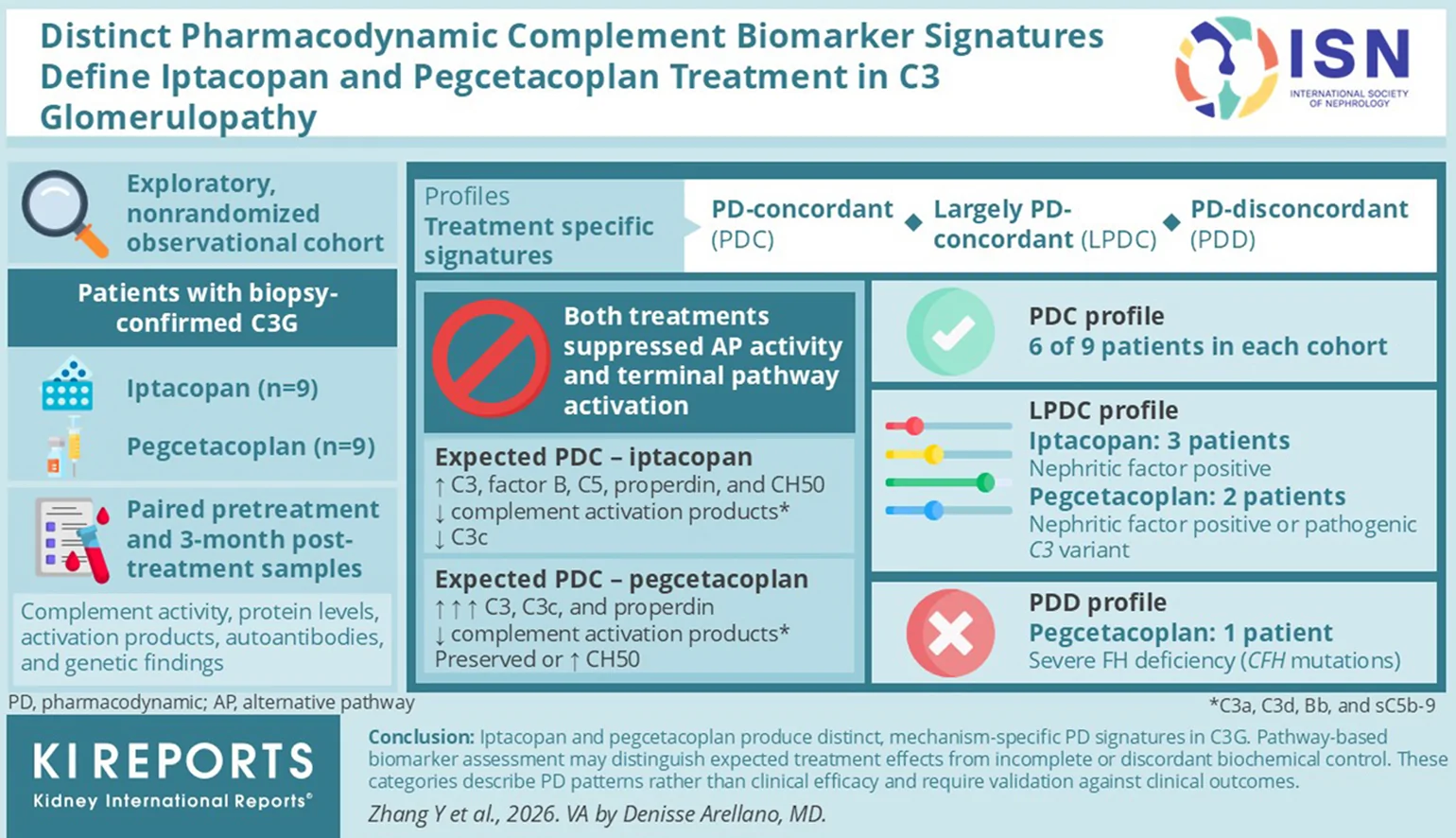

C3G is a rare kidney disease defined by dominant glomerular C3 deposition and AP dysregulation. Persistent AP activation leads to C3 consumption, generation of C3 activation fragments, terminal pathway activation, and chronic glomerular injury.^1^, ^2^, ^3^ Disease drivers are heterogeneous and include nephritic factors (Nefs), autoantibodies to complement regulators, complement gene variants, and combinations of these abnormalities.^4^, ^5^, ^6^, ^7^, ^8^, ^9^

Therapeutic strategies increasingly target the AP at multiple levels of the complement cascade.^10^ Iptacopan inhibits factor B (FB), thereby blocking AP C3 convertase formation and amplification.^11^ Pegcetacoplan binds C3/C3b and inhibits C3-dependent amplification and downstream complement activation.^12^ Clinical studies have reported benefits of both proximal inhibitors in C3G. Iptacopan has been evaluated in an open-label phase 2 study and in the randomized phase 3 APPEAR-C3G trial.^13^, ^14^, ^15^ Pegcetacoplan has been evaluated in a phase 2 study of complement-mediated glomerular diseases and in the randomized phase 3 VALIANT trial in C3G and primary immune-complex MPGN.^12^^,^^16^^,^^17^ These studies were not designed to compare mechanism-specific, multi-analyte PD biomarker profiles at the patient level.

The interpretation of complement biomarkers differs before and during treatment with complement-targeting agents. In untreated C3G, low C3, elevated C3 fragments, increased Ba/Bb, and elevated sC5b-9 generally support ongoing complement activation. During AP-specific complement inhibition, however, these biomarkers must be interpreted in the context of the treatment mechanism.

Because iptacopan and pegcetacoplan inhibit the complement cascade at different levels, their expected on-treatment biomarker patterns also differ. In general, FB inhibition is expected to reduce AP convertase formation and complement consumption, whereas C3/C3b inhibition is expected to cause accumulation of circulating C3-related proteins while limiting downstream activation.^16^^,^^18^

Individual biomarkers therefore should not be interpreted in isolation. We analyzed on-treatment biomarker responses based on 4 biologically related axes: C3 response; AP amplification and factor B turnover; terminal pathway control; and functional pathway activity (Figure 1a). These axes provide a common framework for interpreting the distinct PD signatures of FB inhibition with iptacopan and C3/C3b inhibition with pegcetacoplan. The expected treatment-specific patterns are summarized in Table 1, and the patient-level classification approach is shown in Figure 1b. Using this framework, we performed an exploratory paired-sample PD study in patients with biopsy-confirmed C3G treated with iptacopan or pegcetacoplan. The study was not designed as a head-to-head efficacy comparison. Instead, we sought to define mechanism-specific biomarker signatures and develop a pathway-based framework for classifying patient-level profiles as PDC, LPDC, or PDD.

**Figure 1:**
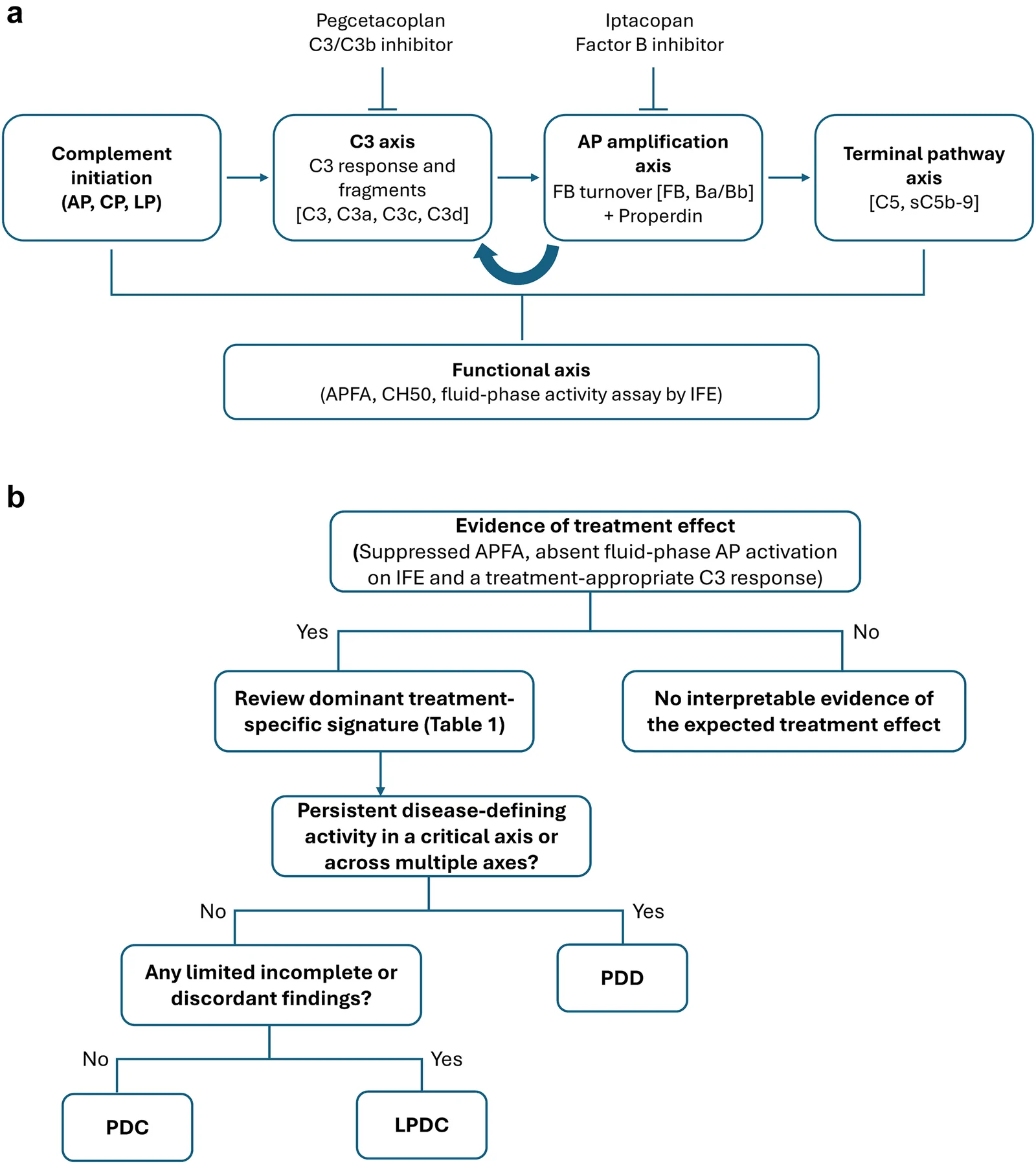
Pathway-based organization and patient-level interpretation of complement biomarkers during proximal complement inhibition. (a) Complement biomarkers are organized into 4 biologically related axes: C3 response, alternative pathway amplification and factor B turnover, terminal pathway control, and functional pathway activity. Pegcetacoplan inhibits C3/C3b, whereas iptacopan inhibits factor B. The curved arrow represents the alternative pathway amplification loop feeding back into C3 activation. (b) Patient-level classification of on-treatment pharmacodynamic biomarker profiles. LPDC, largely PD-concordant; PDC, PD-concordant; PDD, PD-discordant.

**Table 1:** Expected on-treatment pharmacodynamic biomarker patterns during factor B and C3/C3b inhibition

Pathway axis	Biomarker	Iptacopan, factor B inhibitor	Pegcetacoplan, C3/C3b inhibitor
Axis 1: C3 response	Total C3	Restores toward the normal range, often low-normal	Rises markedly, often to supranormal levels
	C3-fragment response	C3a and C3d decrease; C3c decreases or is controlled	C3a and C3d decrease; C3c increases and should be interpreted in the context of the complete treatment-specific profile
Axis 2: AP amplification and FB turnover	Factor B, Ba and Bb	FB is preserved or increases; Ba/Bb decrease or controlled	FB is generally preserved; Ba/Bb decrease or controlled
	Properdin	Preserved or increased	Increases, often markedly
Axis 3: Terminal pathway control	C5 and sC5b-9	C5 is preserved or increases; sC5b-9 decreases	C5 is generally preserved; sC5b-9 decreases
Axis 4: Functional pathway activity	APFA	Suppressed or near absent	Suppressed or near absent
	CH50	Increases or recovers	Generally preserved or increased

AP, alternative pathway; APFA, alternative pathway functional activity; CH50, classical pathway 50% hemolytic activity; FB, factor B; sC5b-9, soluble C5b-9.

## Methods

### Study Design and Participants

We performed an exploratory, nonrandomized observational study of patients with biopsy-confirmed C3G treated with iptacopan or pegcetacoplan. Patients were identified from the Molecular Otolaryngology and Renal Research Laboratories natural history study program between July 2022 and June 2025. All complement testing was performed at Molecular Otolaryngology and Renal Research Laboratories (MORL). The study was approved by the institutional review board of the University of Iowa. Written informed consent was obtained from participants or their legal guardians. The study was conducted in accordance with the Declaration of Helsinki.

No formal sample-size calculation was performed. The study size was determined by the number of eligible participants with paired pretreatment and posttreatment samples available during the study period. Patients who had received C5 inhibitors within 6 months before treatment initiation and patients with monoclonal gammopathy-associated C3G were excluded. Paired pretreatment and approximately 3-month posttreatment serum/plasma samples were analyzed from 18 patients with biopsy-confirmed C3G: 9 treated with iptacopan and 9 treated with pegcetacoplan (Tables 2 and 3; Supplementary Figure S1). Each cohort included 8 patients with C3 glomerulonephritis and 1 patient with dense deposit disease. Disease drivers were heterogeneous. In the iptacopan cohort, 3 patients had no identified driver, 5 had acquired complement autoantibodies such as Nefs, and one was heterozygous for a pathogenic *CFH* variant. In the pegcetacoplan cohort, 1 patient had no identified disease driver, 6 had Nefs, and 2 carried pathogenic variants: Peg#6 was compound heterozygous for 2 pathogenic *CFH* variants, and Peg#9 carried a heterozygous likely pathogenic *C3* variant. An additional posttransplant sample from Peg#6, obtained during subsequent iptacopan treatment, was evaluated descriptively and was not included in the cohort-level iptacopan analyses.

**Table 2:** Paired PD complement biomarker changes during iptacopan treatment

Analyte (reference range)	Pre	Post	% change	*P* value	*q* value
CH50 (41–95 units/ml)	34.1 ± 21.2	66.1 ± 28.8	94%	0.004	0.008
APFA (50%–130%)	24.2 ± 28.4	0.2 ± 0.4	−99%	0.063	0.085
C3 (90–180 mg/dl)	38.2 ± 31.3	103.4 ± 35.3	171%	0.004	0.008
C4 (15–47 mg/dl)	21.4 ± 9.0	22.6 ± 8.5	5%	0.457	0.490
C3a (< 150 μg/l)	124.6 ± 47.6	38.1 ± 20.7	−69%	0.004	0.008
C3c (< 1.5 mg/l)	1.4 ± 0.5	1.0 ± 0.2	−26%	0.039	0.065
C3d (< 8 μg/l)	8.6 ± 1.6	4.7 ± 2.3	−45%	0.004	0.008
Factor B (60–250 mg/l)	67.4 ± 24.7	124.7 ± 66.2	85%	0.008	0.015
Ba (< 1.2 mg/l)	1.8 ± 2.4	0.9 ± 0.7	−51%	0.055	0.082
Bb (< 2.2 mg/l)	2.0 ± 0.8	1.4 ± 0.6	−31%	0.129	0.149
C5 (13.5–27 mg/l)	10.6 ± 4.2	18.8 ± 4.4	77%	0.004	0.008
Properdin (10–33 mg/l)	11.9 ± 4.0	22.1 ± 8.2	86%	0.004	0.008
sC5b-9 (< 0.30 mg/l)	1.4 ± 1.3	0.2 ± 0.2	−84%	0.004	0.008
Factor H (180–420 mg/l)	246.9 ± 77.3	247.7 ± 70.8	0%	0.652	0.652
Factor I (18–44 mg/l)	29.1 ± 6.1	31.3 ± 5.1	8%	0.098	0.122

APFA, alternative pathway functional activity; CH50, classical pathway 50% hemolytic activity; PD, pharmacodynamic; SD, standard deviation.

Values are mean ± SD. Paired sample size was *n* = 9. Values outside the assay measurement range were handled as described in the Statistical analysis section.

**Table 3:** Paired PD complement biomarker changes during pegcetacoplan treatment

Analyte (reference range)	Pre	Post	% change	*P* value	*q* value
CH50 (41–95 units/ml)	35.0 ± 17.8	55.2 ± 11.3	58%	0.039	0.053
APFA (50%–130%)	21.1 ± 27.3	0.0 ± 0.0	−100%	0.063	0.072
C3 (90–180 mg/dl)	37.9 ± 30.0	287.3 ± 71.9	658%	0.004	0.012
C4 (15–47 mg/dl)	23.0 ± 3.5	19.3 ± 5.8	−16%	0.031	0.047
C3a (< 150 μg/l)	165.6 ± 82.2	45.8 ± 32.2	−72%	0.004	0.012
C3c (< 1.5 mg/l)	1.5 ± 0.7	3.9 ± 1.2	159%	0.004	0.012
C3d (< 8 μg/l)	9.7 ± 6.1	5.4 ± 4.6	−45%	0.012	0.022
Factor B (60–250 mg/l)	60.1 ± 27.7	68.0 ± 21.1	13%	0.375	0.375
Ba (< 1.2 mg/l)	2.1 ± 2.4	1.5 ± 2.6	−29%	0.016	0.026
Bb (< 2.2 mg/l)	2.2 ± 1.7	1.2 ± 1.2	−45%	0.012	0.022
C5 (13.5–27 mg/l)	12.5 ± 4.2	14.6 ± 2.8	17%	0.250	0.268
Properdin (10–33 mg/l)	12.4 ± 3.3	50.6 ± 19.0	308%	0.004	0.012
sC5b-9 (< 0.30 mg/l)	0.88 ± 0.84	0.18 ± 0.20	−80%	0.004	0.012
Factor H (180–420 mg/l)	232.7 ± 89.3	190.7 ± 68.6	−18%	0.008	0.020
Factor I (18–44 mg/l)	30.0 ± 4.7	25.8 ± 3.9	−14%	0.020	0.033

APFA, alternative pathway functional activity; CH50, classical pathway 50% hemolytic activity; PD, pharmacodynamic; SD, standard deviation.

Values are mean ± SD. Paired sample size was *n* = 9. Values outside the assay measurement range were handled as described in the Statistical analysis section.

Iptacopan was administered orally at 200 mg twice daily,^19^ and pegcetacoplan was administered subcutaneously at 1080 mg twice weekly.^20^ Standardized clinical outcomes, including proteinuria, eGFR, and repeat-biopsy findings, were not systematically collected; therefore, the analyses focused on laboratory PD patterns.

The paired within-patient design was used to reduce variability arising from differences between patients. Recent C5 inhibitor exposure was excluded to limit potential carryover effects, and monoclonal gammopathy-associated C3G was excluded to reduce etiologic heterogeneity. Treatment assignment was not controlled by this observational study, and no head-to-head clinical efficacy comparison was undertaken.

### Complement Biomarker Measurements

EDTA plasma was used to measure C3, C4, C5, factor B (FB), properdin, C3a, C3c, C3d, Ba, Bb, soluble C5b-9 (sC5b-9), factor H (FH), and factor I (FI).^21^ Serum was used for functional complement assays. Alternative pathway functional activity (APFA) was measured using the Wieslab assay, and all reported clinical CH50 values were measured using a liposome-based functional assay (Thermo Fisher Scientific). The Quidel MicroVue CH50 Eq EIA kit was used only for the pegcetacoplan spiking experiments.

Properdin was measured using a sandwich ELISA that reports total immunoreactive properdin and does not distinguish free properdin from convertase-associated properdin.

Fluid-phase AP activation was evaluated by immunofixation electrophoresis using patient serum mixed with normal human serum under EGTA/MgCl_2_ conditions, which permit AP activation, and EDTA conditions, which suppress complement activation. Nephritic factors, FH and FB autoantibodies, and complement genetic findings were measured and reviewed.^22^^,^^23^

Paired samples from an individual were tested using the same assay platform. Results were interpreted using assay-specific reference ranges.

### Pegcetacoplan Spiking Studies

Pegcetacoplan was added to pooled normal human serum at increasing pegcetacoplan:C3 molar ratios. APFA and CH50 were measured after spiking, and C3 migration was evaluated by immunofixation electrophoresis. To determine whether the slower-migrating C3-reactive band represented pegcetacoplan-bound C3, serum from a pegcetacoplan-treated patient was incubated with increasing concentrations of CP40, a higher-affinity competing compstatin analog.

### Patient-Level PD Categories

Patient-level biomarker profiles were classified as PDC, LPDC, or PDD using a mechanism-informed, pathway-based framework. Classification first required evidence of a treatment-specific PD effect, supported by absent or near-absent APFA when baseline activity was measurable, disappearance of fluid-phase AP activation by IFE, and retention of the dominant treatment-specific signature. Profiles were then evaluated across the 4 pathway axes (Figure 1).

PDC profiles retained the dominant treatment-specific signature without major residual pathway activity. For iptacopan, core features included C3 restoration to the study-specific PD reference level (≥90 mg/dl, corresponding to the lower limit of the reference range), CH50 recovery, preserved or increased FB and C5, and reduced complement activation products. For pegcetacoplan, core features included marked C3 accumulation (≥250 mg/dl), increased C3c and properdin, reduced activation products, and preserved or increased CH50, FB, and C5. The pegcetacoplan C3 threshold of 250 mg/dl was an exploratory, study-specific threshold selected after review of the on-treatment C3 distributions to prompt broader pathway assessment. It did not determine classification in isolation and should not be interpreted as a validated clinical-response threshold. The expected treatment-specific patterns are summarized in Table 1.

LPDC profiles retained evidence of treatment effect and the dominant treatment-specific signature but showed limited, incomplete, or discordant findings within one or more pathway axes. These included incomplete C3 restoration, persistent mild elevation of selected activation products, lack of expected recovery of complement proteins, or functional discordance. During pegcetacoplan treatment, C3 below 250 mg/dl prompted broader review only when accompanied by evidence of residual activation, consumption, or functional abnormality.

PDD profiles showed persistent disease-defining activity within a critical pathway axis or abnormalities across multiple axes despite evidence of a PD effect.

### Statistical Analysis

Continuous biomarker values were analyzed on their original measurement scales. For results below or above an assay’s reportable range, the corresponding lower or upper reportable limit was used in descriptive summaries, percent-change calculations, and paired statistical tests. All participants had paired values for every reported analyte. No biomarker values were missing, and no imputation was performed.

Within each treatment cohort, paired pretreatment and posttreatment biomarker values were compared using exact 2-sided Wilcoxon signed-rank tests, with zero pre/post differences omitted from signed-rank statistics. ***P*** values were adjusted within each cohort using the Benjamini-Hochberg false-discovery-rate procedure. After adjustment for multiple comparisons, statistical significance was defined as ***q*** < 0.05. Nominal ***P*** values are also reported for descriptive purposes. Percent change in Tables 2 and 3 was calculated as [(post-treatment mean − pretreatment mean)/pretreatment mean] x 100. No sensitivity analyses were prespecified or performed.

## Results

### Complement Pathway Activity Under Treatment

APFA decreased after treatment in both cohorts, although absent or near-absent baseline APFA in several patients created a floor effect that limited interpretation (Figure 2a). CH50 increased during iptacopan treatment and was generally preserved or increased during pegcetacoplan treatment (Figure 2b). The increase in CH50 during iptacopan treatment was consistent with reduced C3 consumption and improved functional activity of the classical complement pathway. With pegcetacoplan treatment, preserved CH50 required assay-specific interpretation and was evaluated further in spiking studies. After treatment, immunofixation studies showed disappearance of fluid-phase C3 activation products under EGTA/MgCl_2_ conditions, supporting suppression of AP-driven C3 activation in both cohorts (Figure 2c).

**Figure 2:**
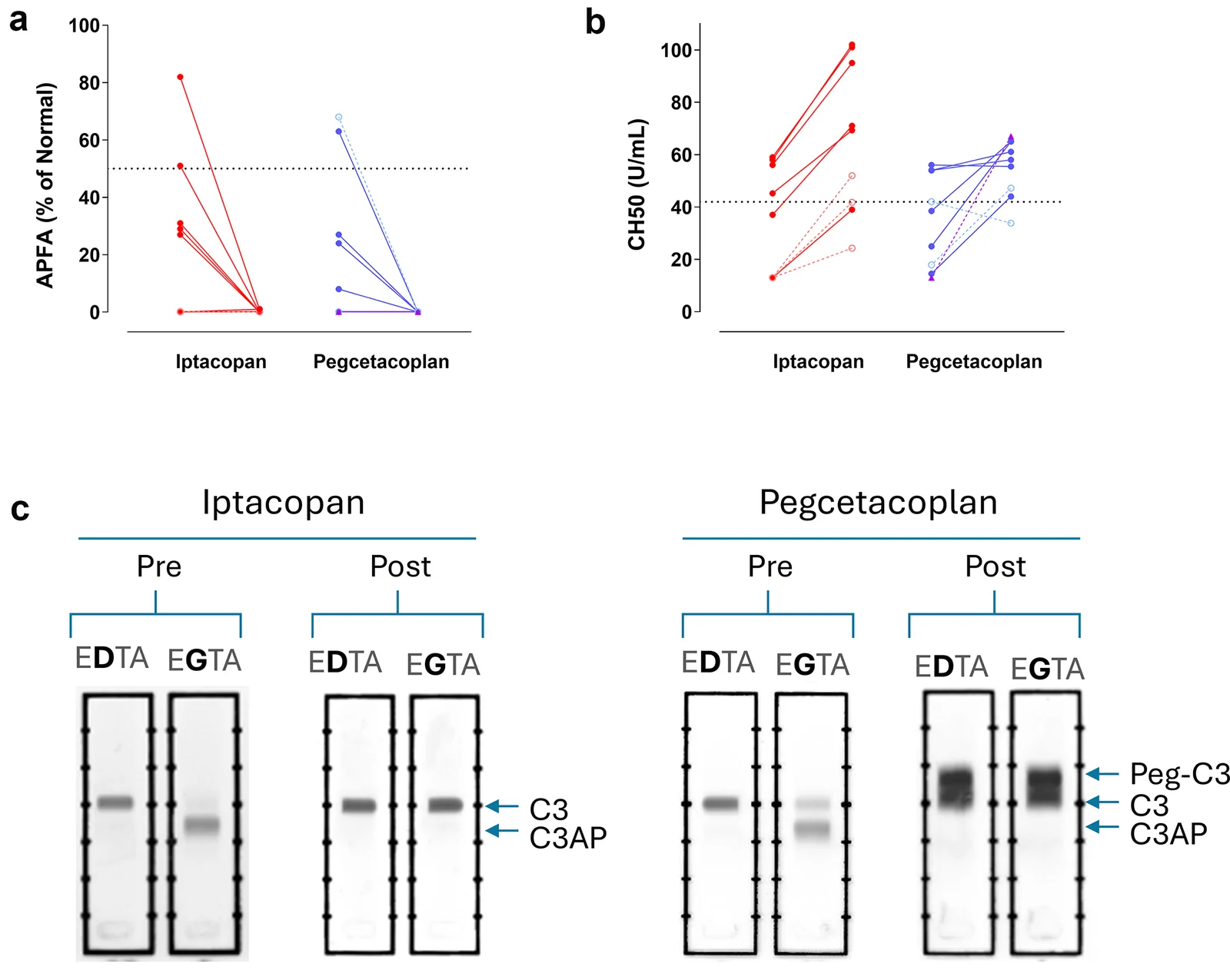
Pathway activity before and after proximal complement inhibition with iptacopan or pegcetacoplan. (a) Alternative pathway activity (APFA) was suppressed after treatment in both cohorts. Several patients had absent or near-absent APFA at baseline, creating a floor effect. (b) Classical pathway activity (CH50) increased after iptacopan and was generally preserved or increased after pegcetacoplan. With iptacopan, CH50 recovery is consistent with AP blockade and recovery of circulating C3. With pegcetacoplan, preserved or increased CH50 should not be interpreted in isolation as absence of target engagement. (c) Immunofixation electrophoresis of patient serum mixed 1:1 with normal human serum under EDTA or EGTA/MgCl_2_ conditions. EDTA suppresses complement activation, whereas EGTA/MgCl_2_ permits AP activation. At baseline, C3 activation products (C3AP) were detectable under EGTA/MgCl_2_ conditions. After treatment, these products disappeared in both treatment groups, consistent with suppression of fluid-phase AP activation. In pegcetacoplan-treated samples, a slower-migrating C3-reactive band was observed, compatible with pegcetacoplan-bound C3 (Peg-C3). Filled circles with solid lines = PDC; open circles with dashed lines = LPDC; triangles = PDD. EDTA, ethylenediaminetetraacetic acid; EGTA, ethylene glycol tetraacetic acid; LPDC, largely PD-concordant; PDC, PD-concordant; PDD, PD-discordant.

### Pegcetacoplan Spiking Studies and CH50 Interpretation

Pegcetacoplan spiking studies clarified the interpretation of CH50 during C3 inhibition (Figure 3). Pegcetacoplan produced concentration-dependent suppression of APFA, with complete suppression at approximately a 1:1 pegcetacoplan:C3 molar ratio, whereas CH50 decreased but remained detectable even in the presence of excess pegcetacoplan (Figure 3a). Immunofixation electrophoresis showed both free C3 and a slower-migrating C3-reactive band consistent with a pegcetacoplan-C3 complex (Figure 3b). Addition of CP40 reduced the complex-associated band and increased free C3, supporting concentration-dependent and reversible pegcetacoplan binding to C3 (Figure 3c). Thus, preserved or restored CH50 during pegcetacoplan treatment may be an expected PD finding and does not necessarily indicate inadequate complement inhibition. An unexpected decline in CH50, or failure of CH50 to recover when baseline activity is low, should prompt broader review of the full pathway pattern.

**Figure 3:**
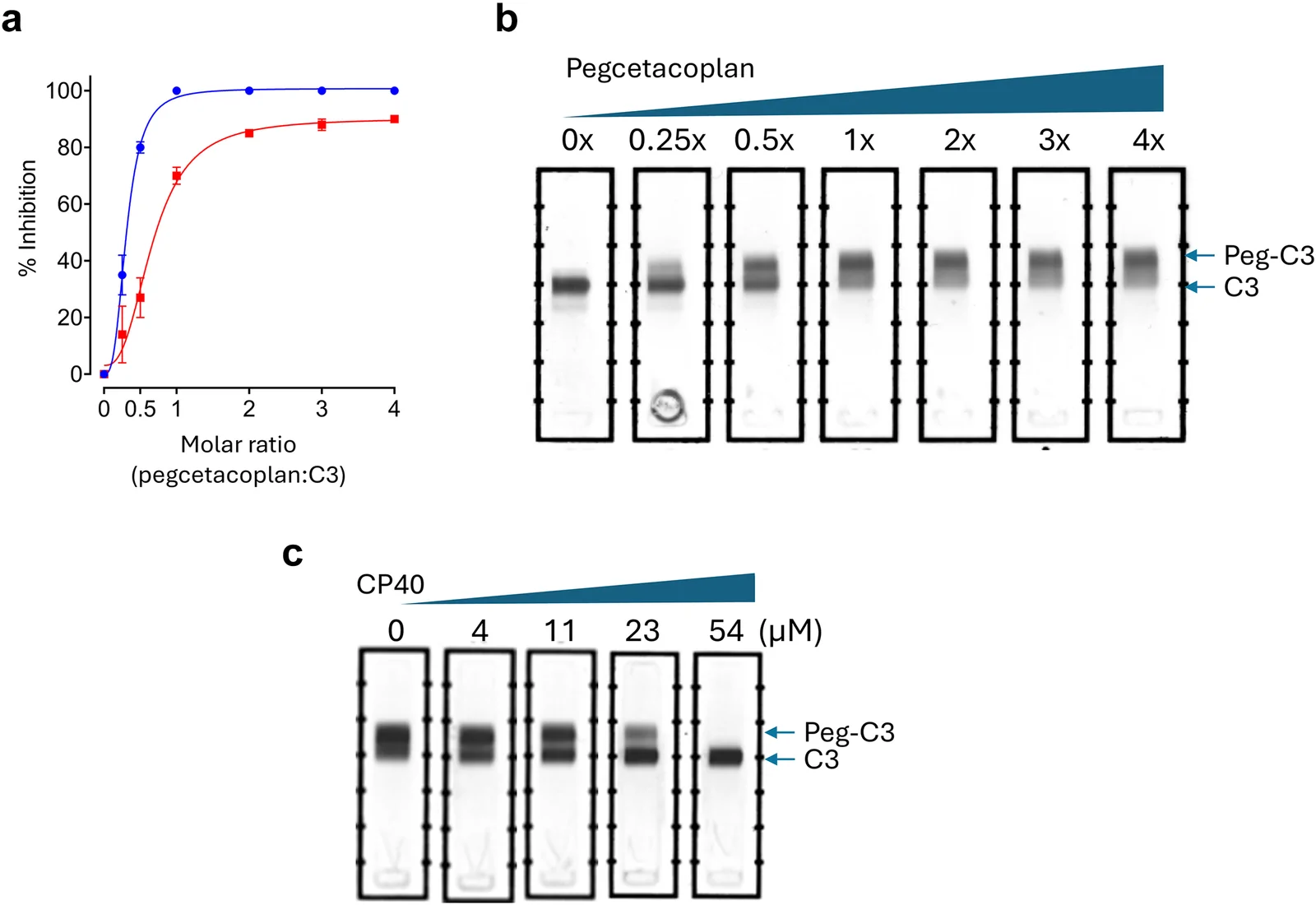
Pegcetacoplan fully suppresses AP activity but incompletely suppresses CH50 *in vitro*. (a) Pegcetacoplan was added to pooled normal human serum at increasing molar ratios relative to C3. AP functional activity (red line), measured with the Wieslab AP assay, was completely suppressed at approximately a 1:1 pegcetacoplan:C3 molar ratio. In contrast, CH50 (blue line) decreased but remained detectable even at a 4:1 ratio, indicating different sensitivities of the two functional assays to pegcetacoplan. (b) C3 immunofixation electrophoresis showed a concentration-dependent increase in a slower-migrating band consistent with pegcetacoplan-bound C3 (Peg-C3) and a corresponding reduction in free C3. Free C3 remained detectable even in the presence of excess pegcetacoplan. Purified C3, shown as the circled dot in the second lane, served as an antibody control. (c) Addition of increasing concentrations of CP40, a competing C3-binding peptide, reduced the pegcetacoplan-C3 band and increased the free-C3 band, supporting concentration-dependent and reversible binding of pegcetacoplan to C3.

### Complement Inhibitor-Specific PD Biomarker Profiles

Six of 9 patients treated with iptacopan showed a PDC profile (Table 2; Figure 4; Supplementary Table S1). After correction for multiple comparisons, CH50, C3, FB, C5, and properdin increased, whereas C3a, C3d, and sC5b-9 decreased. C3 increased into the low-normal range, consistent with reduced AP-mediated consumption. FB and C5 increased, consistent with reduced convertase formation and downstream complement consumption. Ba and Bb decreased numerically but not significantly after correction across all analytes, and FH and FI did not change. Together, the PDC iptacopan signature was characterized by CH50 recovery, C3 restoration to at least the lower limit of normal, preserved or increased FB and C5, reduced or controlled C3 and FB activation products, and reduced sC5b-9.

**Figure 4:**
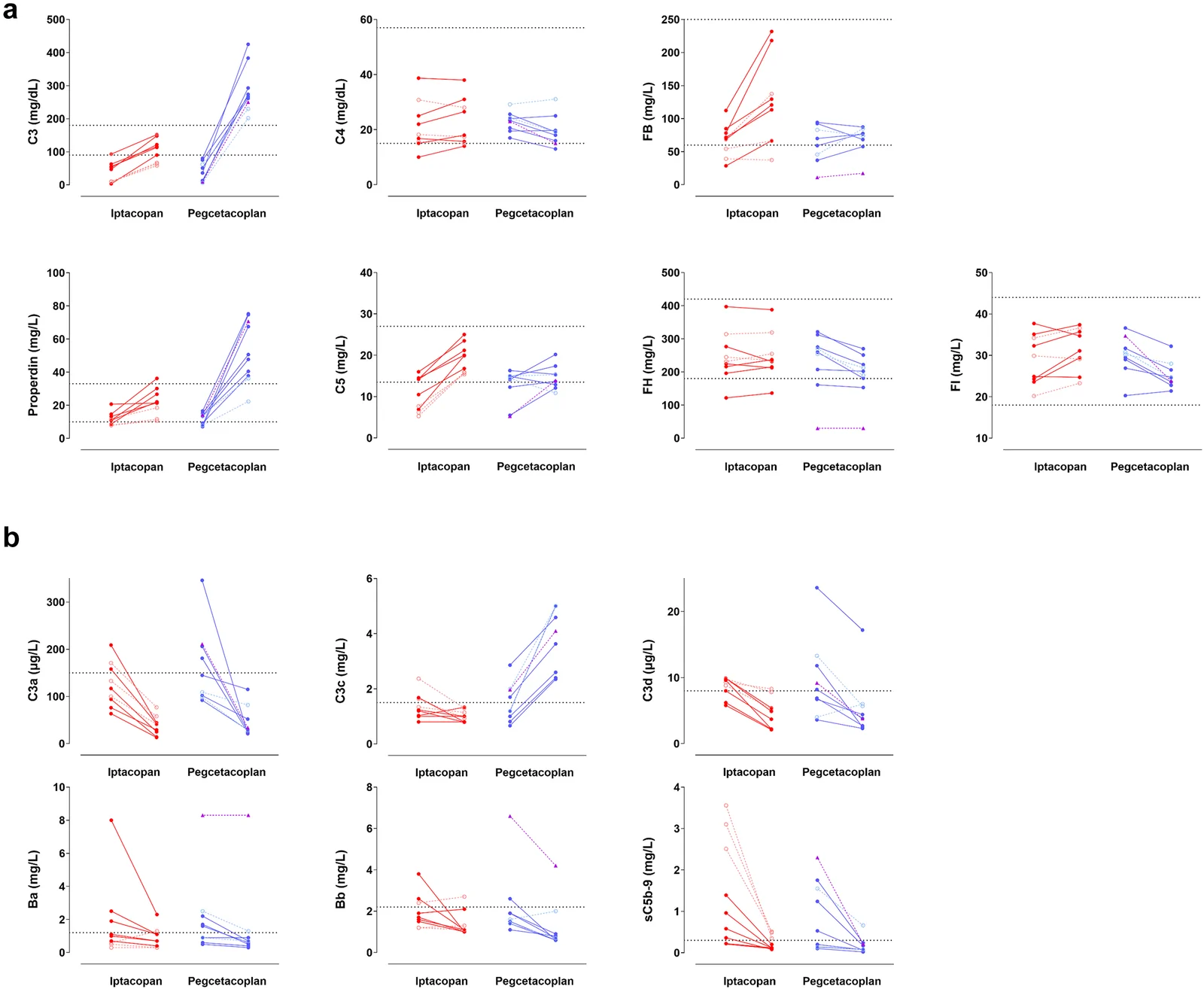
Distinct complement biomarker profiles during iptacopan and pegcetacoplan treatment. Paired pre- and posttreatment complement biomarker values in patients treated with iptacopan or pegcetacoplan. Each connected pair represents 1 patient. (a) Complement proteins, C3, C4, C5, FB, properdin, and fluid-phase regulators, FH and FI. (b) Complement activation products, C3a, C3c, C3d, Ba, Bb, soluble C5b-9 (sC5b-9). Paired changes illustrate the distinct treatment-specific profiles summarized in Table 1 and quantified in Tables 2 and 3. Dashed horizontal lines indicate normal reference limits where available. Filled circles with solid lines = PDC; open circles with dashed lines = LPDC; triangles = PDD.

Six of 9 patients treated with pegcetacoplan showed a distinct PDC C3/C3b-inhibitor profile (Table 3; Figure 4; Supplementary Table S1). After correction for multiple comparisons, C3, C3c, and properdin increased, whereas C3a, C3d, Ba, Bb, sC5b-9, C4, FH, and FI decreased. The reductions in C4, FH, and FI were not considered defining components of the treatment-specific signature, and their biological significance requires confirmation in larger cohorts. C5 and FB did not change. APFA was suppressed after treatment, but the change did not remain statistically significant after multiple-testing correction because many baseline values were already near the assay floor. Together, the PDC pegcetacoplan signature was characterized by C3 at or above the treatment-specific reference level of 250 mg/dl, increased C3c and properdin, reduced or controlled C3a, C3d, Ba/Bb, and sC5b-9, and preserved or increased CH50.

### Patient-Level LPDC and PDD Profiles

After defining the treatment-specific PDC signatures, we reviewed patient-level profiles for LPDC or PDD classification using the overall pattern rather than a single biomarker (Table 4). C3 below the treatment-specific reference level (90 mg/dl for iptacopan and 250 mg/dl for pegcetacoplan) prompted broader PD review, particularly when accompanied by persistent C3d, Ba/Bb, sC5b-9, APFA, or CH50/C5 abnormalities. Review was also triggered when disease-defining activity persisted within a critical pathway axis despite C3 restoration.

**Table 4:** Patient-level PD profile classification during proximal complement inhibition

Treatment PD profile	Dominant pattern (Table 1) retained?	*n*	Patients	Main deviating axis
Iptacopan PD-concordant	Yes	6	Ipt#1, Ipt#2, Ipt#3, Ipt#6, Ipt#8, Ipt#9	None
Iptacopan Largely PD-concordant	Yes, with limited deviation	3	Ipt#4, Ipt#5, Ipt#7	Axis 2: incomplete FB recovery in Ipt#4; Axis 3: residual sC5b-9 elevation in Ipt#5 and Ipt#7
Iptacopan PD-discordant		0	None	Not observed^a^
Pegcetacoplan PD-concordant	Yes	6	Peg#1, Peg#2, Peg#3, Peg#4, Peg#5, Peg#8	None
Pegcetacoplan Largely PD-concordant	Yes, with limited deviation	2	Peg#7, Peg#9	Peg#7: limited deviations across Axes 1–3; Peg#9: limited deviations across Axes 1, 3, and 4
Pegcetacoplan PD-discordant	Critical axis persists	1	Peg#6	Axis 2: persistent FB depletion and Ba/Bb elevation despite C3 and terminal pathway control

FB, factor B; LPDC, largely pharmacodynamic-concordant; PD, pharmacodynamic; PDC, pharmacodynamic-concordant; PDD, pharmacodynamic-discordant.

PDC indicates preservation of the dominant treatment-specific signature; LPDC indicates preservation of the dominant signature with limited deviation; PDD indicates persistent activity in a critical axis, or discordance across multiple axes.

a The posttransplant iptacopan sample from Peg#6 was evaluated descriptively and classified as PDD but was not included in the iptacopan cohort counts.

In the iptacopan cohort, 3 patients showed LPDC profiles, and all were C5Nef-positive (Table 4). In all 3, C3 increased but did not normalize, prompting broader review. Ipt#4, who had C4- and C5Nefs, showed APFA suppression and reductions in C3a, C3d, and sC5b-9, but FB failed to recover. Ipt#5 and Ipt#7, both positive only for C5Nefs, retained the core iptacopan response but showed mild residual sC5b-9 elevation.

In the pegcetacoplan cohort, 2 patients showed LPDC profiles (Table 4). Peg#7, who was strongly positive for C3-, C4-, and C5Nefs, showed increased C3, C3c, and properdin and reduced Ba and C3d. However, C3 remained below the treatment-specific reference level, C5 decreased, Bb increased, and sC5b-9 suppression was incomplete.

Peg#9, who carried a heterozygous likely pathogenic *C3* variant (Table 5), showed suppression of APFA, an increase in C3 from 62 to 230 mg/dl, reduction of C3a and Ba/Bb, and increases in C3c and properdin. However, CH50 decreased, C3d increased, and C5 remained essentially unchanged. The profile was therefore classified as LPDC because the dominant pegcetacoplan-associated signature was retained but limited deviations persisted across the C3, terminal pathway, and functional axes. The available data do not establish whether the C3 variant contributed directly to this profile.

**Table 5:** Cohort and sample characteristics

Characteristic	Iptacopan	Pegcetacoplan
Patients, *n*	9	9
Age range, yrs	19–52	13–36
Sex, male/female	4/5	4/5
C3GN/DDD, *n*	8/1	8/1
Posttreatment sampling interval	∼ 3 mos	∼ 3 mos
Dose	200 mg twice daily	1080 mg twice weekly
Disease drivers		
Unknown, *n*	3	1
Acquired, *n*	5	6
C3Nefs only, *n*	1	3
C4Nefs only, *n*	0	1
C5Nefs only, *n*	2	0
C4/C5Nefs, *n*	1	1
C3/C4/C5Nefs, *n*	0	1
FHAA, *n*	1	0
Pathogenic or likely pathogenic variants		
*CFH* variant, (*n*)	*CFH* p.Cys597Ter (1)	*CFH* p.Tyr182Ter; p.Thr430Pro (1)^a^
*C3* variant, (*n*)	0	*C3* p.Ser1150Trp (1)

C3GN, C3 glomerulonephritis; DDD, dense deposit disease; FHAA, factor H autoantibodies; Nefs, nephritic factors.

a Compound heterozygote.

Peg#6 showed the most biologically discordant PD profile during pegcetacoplan treatment (Tables 4 and 6). This patient carried compound heterozygous pathogenic *CFH* variants and had persistently undetectable circulating FH. A clear PD effect was present, with CH50 recovery, marked C3 restoration, reductions in C3a and C3d, restoration of C5, increased C3c and properdin, and marked suppression of sC5b-9. However, the AP amplification and FB turnover axis remained abnormal: FB remained profoundly depleted despite partial improvement, Ba remained above the assay range, and Bb remained elevated despite partial reduction. The profile was classified as PDD because severe FH deficiency was associated with persistent upstream FB turnover despite downstream C3-fragment and terminal pathway control, not because pegcetacoplan lacked PD effect.

**Table 6:** Biomarker profile in Peg#6 during pegcetacoplan treatment and after post-transplant switch to iptacopan

Biomarker	Pre-pegcetacoplan	On pegcetacoplan	Post-transplant on iptacopan	Normal reference range
CH50	13	67	98	41–95 units/ml
APFA	0	0	0	50%–130%
C3	9	250	96	90–180 mg/dl
C4	23	15	17	15–47 mg/dl
C3a	211	34	87	< 150 μg/l
C3c	1.97	4.1	1.3	< 1.5 mg/l
C3d	9.2	3.8	< 2.3	< 8 μg/l
FB	11	17	33	60–250 mg/l
Ba	> 8.2	> 8.2	3.2	< 1.2 mg/l
Bb	6.62	4.2	4.7	< 2.2 mg/l
C5	5.3	13.9	16.8	13.5–27 mg/l
Properdin	14.2	70.7	29.4	10–33 mg/l
sC5b-9	2.3	0.21	0.18	< 0.30 mg/l
FH	< 34	< 34	< 34	180–420 mg/l
FI	34.7	23.8	31	18–44 mg/l

APFA, alternative pathway functional activity; CH50, classical pathway 50% hemolytic activity; FB, factor B; FH, factor H; FI, factor I.

After kidney transplantation, Peg#6 was switched to iptacopan. The post-transplant sample showed preserved CH50, absent APFA, restored C3, controlled C3d, preserved C5, and suppressed sC5b-9. FH remained undetectable. FB increased from 17 to 33 mg/l but remained below the reference range, and Ba and Bb remained elevated. Thus, downstream pathway control improved, but persistent dysregulation of the FB/Ba/Bb axis in the setting of profound FH deficiency supported continued classification as PDD.

This case suggests that FH deficiency may allow persistent AP turnover during either treatment, despite pharmacologic inhibition at different points in the pathway. FH normally accelerates decay of the AP C3 convertase and supports FI–mediated inactivation of C3b. When FH is severely deficient, spontaneous C3 hydrolysis (“tick-over”) may continue to drive convertase formation, resulting in factor B consumption and generation of Ba and Bb. Consequently, abnormalities in FB, Ba, and Bb may persist even when C3-fragment and terminal pathway markers are controlled. This case also supports longitudinal, pathway-based monitoring, particularly after transplantation or a change in treatment.

## Discussion

In this exploratory paired-sample study, iptacopan and pegcetacoplan both suppressed AP activity and reduced terminal pathway activation in C3G, but they produced distinct mechanism-specific PD biomarker profiles (Figures 1 and 4). The central finding is not that either agent produced a superior biomarker response. Rather, each agent generated a coherent treatment-specific signature that must be interpreted according to its site of action.

The iptacopan signature was consistent with factor B inhibition: C3 and CH50 recovered, FB and C5 were preserved or increased, and C3 activation fragments and sC5b-9 decreased. Limited C3 recovery, persistent activation products, or continued FB consumption were most informative when they occurred together within related axes rather than as isolated abnormalities.

The pegcetacoplan signature was consistent with C3/C3b inhibition: C3 increased to supranormal levels, C3c and properdin increased, C3a, C3d, Bb, and sC5b-9 decreased, and CH50 was generally preserved or increased. Although this pattern may appear counterintuitive, high C3, increased C3c and properdin, and measurable CH50 are compatible with a PD effect when AP functional activity, C3 activation products, factor B split products, and sC5b-9 are suppressed. Persistent abnormalities across several of these markers, or an unexpected decline in CH50, should prompt broader pathway review.

C3 was an important reference biomarker in both cohorts, but classification required concordant changes across the 4 pathway axes. For iptacopan, the restoration of C3 and FB toward normal ranges was weighted heavily, as FB inhibition directly reduces AP C3 convertase activity and subsequent C3 consumption. For pegcetacoplan, supranormal C3 accumulation was expected because of direct C3/C3b blockade. In both settings, a single abnormal biomarker was insufficient for LPDC or PDD classification. Instead, classification weight was given to unexpected abnormalities that clustered within related biological axes.

Disease-driver information helped interpret PD heterogeneity but did not define treatment-selection rules. PDC and LPDC profiles occurred across acquired and genetic driver categories.

All 3 iptacopan LPDC profiles occurred in patients with C5Nefs. Ipt#4, who had both C4Nefs and C5Nefs, showed suppression of APFA and reductions in C3a, C3d, and sC5b-9, but C3 restoration was incomplete and factor B failed to recover. Ipt#5 and Ipt#7, who had C5Nefs without another identified driver, retained the dominant iptacopan-associated signature but showed incomplete C3 restoration and mild residual sC5b-9 elevation. These observations suggest that C5Nef-containing profiles may provide biological context for residual terminal pathway abnormalities during factor B inhibition. However, the small number of patients and lack of clinical-outcome correlations preclude a causal conclusion.

Peg#7, who was strongly positive for C3-, C4-, and C5Nefs, showed an LPDC profile with limited deviations across the C3, AP amplification, and terminal pathway axes. The presence of multiple Nefs provides biological context for incomplete biochemical control, but this single observation does not establish causality.

Peg#9 carried a heterozygous likely pathogenic *C3* variant in the thioester-containing domain and showed an LPDC profile during pegcetacoplan treatment, with limited discordance across several pathway axes. *C3* variants may alter AP regulation through variant-specific mechanisms. Selected gain-of-function variants within the C3 MG-ring have recently been reported to reduce pegcetacoplan binding in paroxysmal nocturnal hemoglobinuria.^24^ However, these effects appear to be domain- and variant-specific. Variants outside the MG-ring could influence the expected PD profile through mechanisms other than impaired pegcetacoplan binding. Because direct pegcetacoplan-binding and variant-specific functional studies were not performed, we cannot determine whether *C3* p.Ser1150Trp contributed to the observed profile. This case supports integrating genetic findings with functional and biochemical measurements rather than inferring treatment resistance from the presence of a *C3* variant alone.

Peg#6 demonstrated that a clear proximal PD effect and downstream pathway control can coexist with persistent disease-defining FB turnover in severe FH deficiency. The continued FB/Ba/Bb abnormality during both treatments supports classification based on related pathway axes rather than C3 or terminal pathway markers alone.

The clinical implications of this study are interpretive rather than therapeutic. PDC, LPDC, and PDD describe biochemical pathway concordance, not clinical efficacy, drug superiority, or treatment-selection thresholds. The framework is intended to distinguish expected treatment-specific effects from incomplete or discordant biochemical control and requires correlation with proteinuria, eGFR, histology, and other clinical outcomes.

The study was limited by its small, nonrandomized cohort, uncontrolled treatment assignment, and inclusion of only patients with paired samples, which may limit representativeness. Clinical outcomes, adherence, pharmacokinetic data, and sampling relative to the most recent dose were not systematically available. Use of assay-boundary values for censored measurements may also have influenced some paired changes. Because the classification framework was developed and applied in the same specialized cohort, it may be overfit and may not generalize across populations, sampling schedules, or laboratory platforms. Independent prospective validation with standardized sampling is therefore required.

In conclusion, iptacopan and pegcetacoplan suppress complement activation in C3G but generate distinct PD biomarker signatures. A mechanism-informed, pathway-based approach incorporating CH50, APFA, treatment-specific C3 responses, C3 activation fragments, FB/Ba/Bb, properdin, C5, and sC5b-9 may help distinguish expected treatment-specific effects from incomplete or discordant biochemical pathway control. Future longitudinal studies incorporating clinical outcomes, repeat histology, treatment-switch data, and standardized sampling relative to drug dosing are needed to determine whether these PD profiles predict renal response, relapse, transplant recurrence, infection risk, long-term kidney outcomes, or benefit from switching between proximal complement inhibitors.

## Disclosure

RJHS and CMN serve as Director and Associate Director, respectively, of the Molecular Otolaryngology and Renal Research Laboratories. CMN serves on advisory boards for Novartis, Apellis, Vertex, and Alexion; participates as a site investigator for Novartis, Apellis, BioCryst, and Retrophin; is a member of the data safety monitoring board for Kira; and serves as Chair of the data safety monitoring board for FIT4KID. RJHS serves on the advisory board for Novartis. All other authors declared no competing interests.

## Supplementary Material

Supplementary File (PDF) **Figure S1.** Patient and sample selection for paired biomarker analysis. **Table S1.** Raw paired patient-level biomarker values. STROBE Statement.

## Footnotes

Supplementary File (PDF)
**Figure S1.** Patient and sample selection for paired biomarker analysis. **Table S1.** Raw paired patient-level biomarker values. STROBE Statement.
