# Supplementary material for: Distinct Pharmacodynamic Complement Biomarker Signatures Define Iptacopan and Pegcetacoplan Treatment in C3 Glomerulopathy
Source: Kidney Int Rep. 2026 Sep 18;11(12):107087. doi: 10.1016/j.ekir.2026.107087 (PMC13678011; doi:10.1016/j.ekir.2026.107087)
Supplement: Supplementary file 1 [file mmc1.pdf]

Supplementary Figure S1

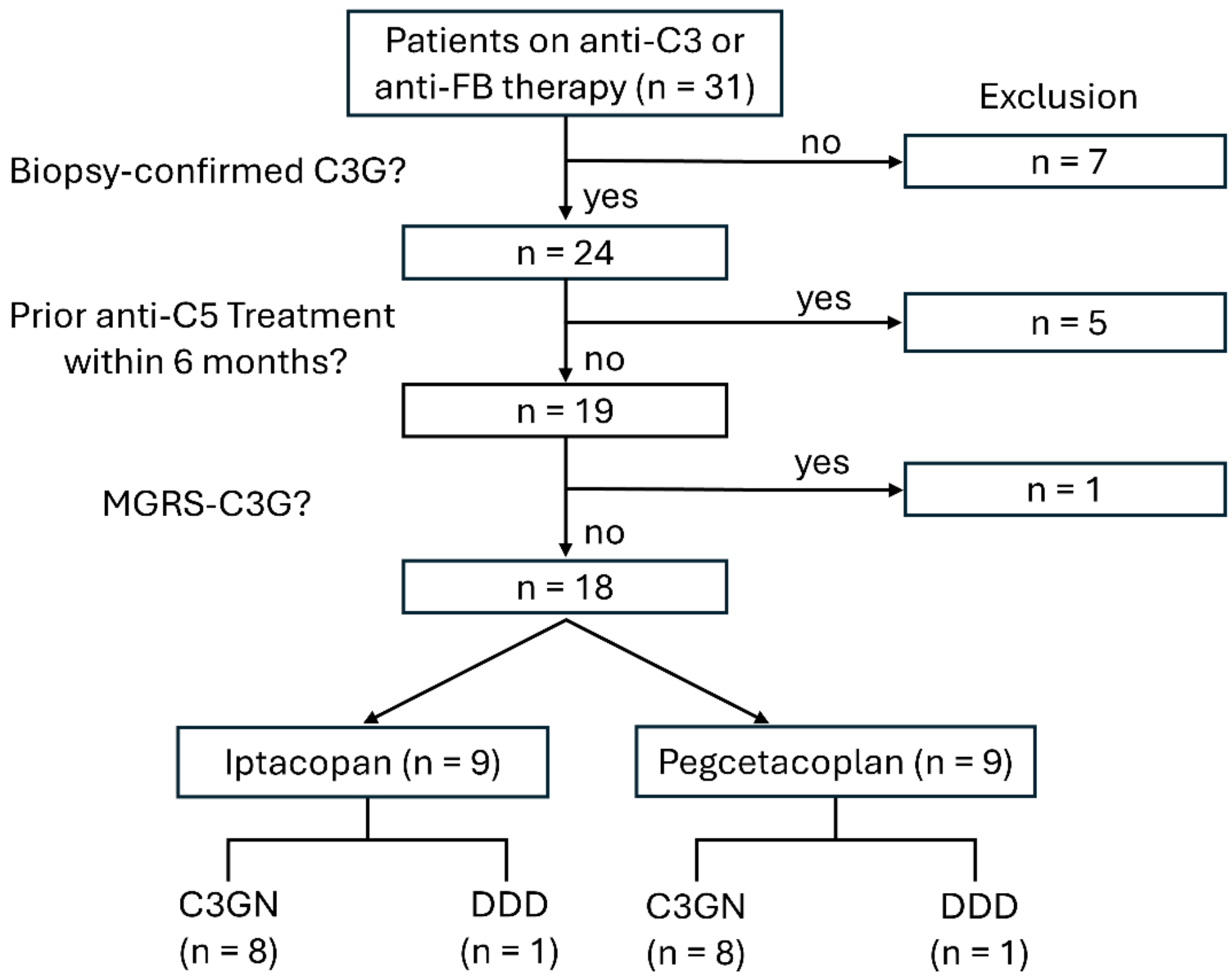

Supplementary Figure S1. Patient and sample selection for paired biomarker analysis

Flowchart showing inclusion and exclusion of samples for this exploratory pharmacodynamic study. Patients treated with a C3 or factor B inhibitor were screened for biopsy-confirmed C3G and availability of paired serum/ plasma samples collected before treatment and ~ 3 months after initiation of iptacopan or pegcetacoplan. Patients who had received C5 inhibitors within 6 months before treatment initiation and those with monoclonal gammopathy–associated C3G (MGRS) were excluded. The final cohort included 9 iptacopan-treated patients and 9 pegcetacoplan-treated patients. In each treatment cohort, 8 patients had C3GN and 1 had DDD.

Supplementary Table S1. Raw paired patient-level biomarker values

Iptacopan

ID	Disease Driver	CH50	APFA	C3	C4	C3a	C3c	C3d	FB	Ba	Bb	C5	P	sC5b-9	FH	FI	PD Class
Ipt#1	Unknown	45.2→ 69.3	29→ 0	55→ 113	17→ 16	63.5→ 13	0.83→ 0.79	8.0→ 3.7	69→ 121	0.95→ 0.66	1.49→ 0.96	14.2→ 21.2	10.9→ 22.1	0.36→ 0.08	225→ 213	23.6→ 29.4	PDC
Ipt#2	FHAA	58→ >102	29→ 0	63→ 116	10→ 14	94→ 14	1.01→ 0.99	5.8→ 2.3	85→ 130	1.09→ 0.70	2.58→ 1.10	14.5→ 20.0	8.6→ 26.7	0.22→ 0.12	196→ 215	24.9→ 24.7	PDC
Ipt#3	C3Nefs	<13→ 39	0→ 0	3→ 90	39→ 38	209→ 44	1.03→ 1.33	9.9→ 5.4	29→ 67	>8.0→ 2.33	1.93→ 2.06	6.9→ 19.9	20.7→ 21.3	0.58→ 0.12	276→ 231	32.3→ 35.7	PDC
Ipt#4	C4/C5 Nefs	<12.8→ 24.3	0→ 1	10→ 67	18→ 17	133→ 58	1.64→ 0.93	8.8→ 5.3	39→ 37	0.32→ 0.28	1.16→ 1.08	7.7→ 15.4	8.0→ 11.1	3.56→ 0.35	314→ 319	29.9→ 29.1	LPDC
Ipt#5	C5Nefs	<13→ 52	0→ 0	10→ 65	31→ 28	171→ 77	2.37→ 1.30	9.3→ 8.3	69→ 138	0.60→ 1.26	2.35→ 2.68	6.2→ 15.9	11.9→ 18.5	2.51→ 0.51	245→ 235	34.2→ 36.6	LPDC
Ipt#6	Unknown	56.0→ >94.5	27→ 0	53→ 122	22→ 26	158→ 42	1.20→ 0.79	9.9→ 4.9	112→ 232	1.86→ 1.11	1.64→ 1.09	14.3→ 25.0	13.3→ 21.5	1.39→ 0.20	397→ 388	35.1→ 37.4	PDC
Ipt#7	C5Nefs	13.0→ 41.8	0→ 0	10→ 58	16→ 15	100→ 41	1.34→ 1.14	9.8→ 7.8	54→ 66	0.48→ 0.31	1.16→ 1.34	5.3→ 11.1	7.9→ 11.1	3.10→ 0.49	231→ 255	20.2→ 23.4	LPDC
Ipt#8	Unknown	37→ 71	51→ 0	47→ 148	15→ 18	76→ 29	1.68→ 0.80	9.6→ 2.3	72→ 113	0.70→ 0.40	1.70→ 1.00	10.5→ 16.8	10.9→ 30.1	0.96→ 0.11	216→ 237	24.3→ 31.1	PDC
Ipt#9	CFH variant, het	59→ >101	82→ 1	93→ 152	25→ 31	117→ 25	1.23→ 1.00	6.2→ 2.3	78→ 218	2.51→ 1.10	3.78→ 1.00	16.0→ 23.5	14.6→ 36.2	0.21→ 0.10	122→ 136	37.7→ 34.7	PDC

Values are presented as pretreatment → posttreatment

Supplementary Table S1. Raw paired patient-level biomarker values (continued)

Pegcetacoplan

ID	Disease Driver	CH50	APFA	C3	C4	C3a	C3c	C3d	FB	Ba	Bb	C5	P	sC5b-9	FH	FI	PD Class
Peg#1	C3Nefs	56.0→ 55.5	63→ 0	80→ 383	26→ 19	181→ 24	1.70→ 3.63	8.2→ 3.8	94→ 87	1.58→ 0.57	2.61→ 0.61	16.3→ 15.4	13.4→ 40.5	0.2→ 0.07	312→ 270	31.7→ 26.5	PDC
Peg#2	C4Nefs	14.5→ 44	0→ 0	9→ 293	21→ 16	102→ 52	2.86→ 4.59	11.8→ 2.5	70→ 77	0.59→ 0.38	1.89→ 0.91	5.46→ 12.1	9.2→ 37.8	1.75→ 0.24	260→ 181	26.9→ 24.7	PDC
Peg#3	C3Nefs	38.5→ 65	0→ 0	13→ 274	20→ 20	346→ 21	0.66→ 2.35	6.9→ 2.7	37→ 58	1.71→ 0.54	1.39→ 0.55	14.9→ 12.9	15.2→ 50.6	0.10→ 0.02	207→ 202	29.0→ 22.8	PDC
Peg#4	C3Nefs	54→ 58	27→ 0	75→ 267	17→ 11	206→ 27	1.00→ 2.60	3.6→ <2.3	48→ 68	0.50→ 0.30	1.50→ 0.60	14.8→ 17.4	16.6→ 75.2	0.14→ 0.08	161→ 153	20.3→ 21.4	PDC
Peg#5	C4/C5 Nefs	25→ 65	8→ 0	36→ 425	24→ 25	145→ 115	1.20→ 5.00	23.6→ 17	59→ 78	0.88→ 0.90	1.15→ 0.80	14.1→ 20.2	7.8→ 74.7	1.24→ 0.17	321→ 251	36.6→ 32.2	PDC
Peg#6	CFH variant, comp het	13→ 67	0→ 0	9→ 250	23→ 15	211→ 34	1.97→ 4.10	9.2→ 3.8	11→ 17	>8.2→ >8.2	6.62→ 4.20	5.3→ 13.9	14.2→ 70.7	2.3→ 0.21	<34→ <34	34.7→ 23.8	PDD
Peg#7	C3/C4/C5 Nefs	17.9→ 47.2	0→ 0	6→ 202	29→ 31	109→ 82	1.99→ 5.00	13.3→ 5.7	83→ 74	0.92→ 0.68	1.61→ 2.01	14.4→ 10.9	7.9→ 22.3	1.55→ 0.66	254→ 213	30.8→ 24.6	LPDC
Peg#8	Unknown	54.0→ 61.1	24→ 0	51→ 262	23→ 18	94→ 29	0.81→ 2.40	6.7→ 4.4	93→ 68	2.23→ 0.68	1.85→ 0.67	12.3→ 13.7	13.6→ 47.7	0.53→ 0.06	275→ 222	29.5→ 23.6	PDC
Peg#9	C3 variant, het	42.0→ 33.8	68→ 0	62→ 230	24→ 19	96→ 28	1.19→ 5.00	4.0→ 6.0	46→ 85	2.52→ 1.29	1.43→ 0.69	15.0→ 15.3	13.9→ 36.2	0.13→ 0.08	270→ 190	30.2→ 32.2	LPDC

Values are presented as pretreatment → posttreatment

STROBE Statement—checklist of items that should be included in reports of observational studies

Manuscript: Distinct Pharmacodynamic Complement Biomarker Signatures Define Iptacopan and Pegcetacoplan Treatment in C3 Glomerulopathy

	Item No.	Recommendation	Page No.	Relevant text from manuscript
**Title and abstract**	1	(*a*) Indicate the study's design with a commonly used term in the title or the abstract	p. 1	Abstract Methods: an exploratory, nonrandomized observational cohort study of patients with biopsy-confirmed C3G treated with iptacopan (n = 9) or pegcetacoplan (n = 9), using paired pretreatment and approximately 3-month post-treatment samples.
		(*b*) Provide in the abstract an informative and balanced summary of what was done and what was found	p. 1	Iptacopan and pegcetacoplan produce distinct, mechanism-specific PD signatures in C3G. Pathway-based biomarker assessment may distinguish expected treatment effects from incomplete or discordant biochemical control. These categories describe PD patterns rather than clinical efficacy and require validation against clinical outcomes.
**Introduction**				
Background/rationale	2	Explain the scientific background and rationale for the investigation being reported	pp. 1-2	Disease drivers are heterogeneous and include nephritic factors (Nefs), autoantibodies to complement regulators, complement gene variants, and combinations of these abnormalities.<br>Because iptacopan and pegcetacoplan inhibit the complement cascade at different levels, their expected on-treatment biomarker patterns also differ.
Objectives	3	State specific objectives, including any prespecified hypotheses	p. 2	we sought to define mechanism-specific biomarker signatures and develop a pathway-based framework for classifying patient-level profiles as PD-concordant (PDC), largely PD-concordant (LPDC), or PD-discordant (PDD).
**Methods**				
design	4	Present key elements of study design early in the paper	p. 2	We performed an exploratory, nonrandomized observational study of patients with biopsy-confirmed C3G treated with iptacopan or pegcetacoplan.
Setting	5	Describe the setting, locations, and relevant dates, including periods of recruitment, exposure, follow-up, and data collection	p. 2	Patients were identified from the Molecular Otolaryngology and Renal Research Laboratories (MORL) natural history study program between July 2022 and June 2025. All complement testing was performed at MORL. Treatment exposure and approximately 3-month post-treatment sampling are reported.

Participants	6	(*a*) *Cohort study*—Give the eligibility criteria, and the sources and methods of selection of participants. Describe methods of follow-up	p. 2 + Suppl. Fig. S1	Patients who had received C5 inhibitors within 6 months before treatment initiation and patients with monoclonal gammopathy-associated C3G were excluded. Participant flow is shown in Supplementary Figure 1.
		Case-control study—Give the eligibility criteria, and the sources and methods of case ascertainment and control selection. Give the rationale for the choice of cases and controls<br>*Cross-sectional study*—Give the eligibility criteria, and the sources and methods of selection of participants	Figure 1	
		(*b*) *Cohort study*—For matched studies, give matching criteria and number of exposed and unexposed<br>*Case-control study*—For matched studies, give matching criteria and the number of controls per case	N/A	
Variables	7	Clearly define all outcomes, exposures, predictors, potential confounders, and effect modifiers. Give diagnostic criteria, if applicable	pp. 2-4	Treatment exposure, complement biomarkers, functional assays, nephritic factors, autoantibodies, genetic findings, and the PDC/LPDC/PDD categories are defined in Methods and Table 1.
Data sources/ measurement	8*	For each variable of interest, give sources of data and details of methods of assessment (measurement). Describe comparability of assessment methods if there is more than one group	pp. 2-3	See Methods Complement biomarker measurements section
Bias	9	Describe any efforts to address potential sources of bias	pp. 2 and 10	The paired within-patient design reduced between-patient variability. Recent C5 inhibitor exposure and monoclonal gammopathy-associated C3G were excluded to limit carryover and etiologic heterogeneity. The Discussion addresses selection, treatment-assignment, measurement, and overfitting concerns.
Study size	10	Explain how the study size was arrived at	p.2 + Suppl. Fig. S1	No formal sample-size calculation was performed. The study size was determined by the number of eligible participants with paired pretreatment and post-treatment samples available during the study period. Participant and sample selection are shown in Supplementary Figure 1.

Quantitative variables	11	Explain how quantitative variables were handled in the analyses. If applicable, describe which groupings were chosen and why	p. 5	Continuous biomarkers were analyzed on their original scales. Assayboundary values were substituted for measurements outside reportable ranges. Percent change and laboratory reference ranges are reported..

Statistical methods	12	(*a*) Describe all statistical methods, including those used to control for confounding	p. 5	Within each treatment cohort, paired values were compared using exact two-sided Wilcoxon signed-rank tests, with zero differences omitted. P values were adjusted within cohort using the Benjamini-Hochberg falsediscovery-rate procedure; q<0.05 defined statistical significance. Percent change was calculated from paired values.
		(*b*) Describe any methods used to examine subgroups and interactions	N/A	
		(*c*) Explain how missing data were addressed	p. 5	All participants had paired values for every reported analyte; no biomarker values were missing and no imputation was performed. Values outside reportable ranges were handled using assay-boundary values.
		(*d*) *Cohort study*—If applicable, explain how loss to follow-up was addressed<br>*Case-control study*—If applicable, explain how matching of cases and controls was addressed<br>*Cross-sectional study*—If applicable, describe analytical methods taking account of sampling strategy	N/A	
		(*e*) Describe any sensitivity analyses	p. 5	No sensitivity analyses were prespecified or performed.
**Results**				
Participants	13*	(a) Report numbers of individuals at each stage of study—eg numbers potentially eligible, examined for eligibility, confirmed eligible, included in the study, completing follow-up, and analysed	Supple. Fig. S1	The flow diagram reports 31 patients receiving anti-C3 or anti-FB treatment, 24 with C3G, 23 after excluding monoclonal gammopathy-associated disease, and 18 after excluding recent C5 inhibitor exposure; 9 iptacopan-treated and 9 pegcetacoplan-treated participants were analyzed.
		(b) Give reasons for non-participation at each stage	Supple. Fig. S1	Seven patients without C3G, one with monoclonal gammopathyassociated C3G, and five with C5 inhibitor exposure within 6 months were excluded.
		(c) Consider use of a flow diagram	Supple. Fig. S1	Supplementary Figure 1 provides the participant and sample-selection flow diagram.
Descriptive data	14*	(a) Give characteristics of study participants (eg demographic, clinical, social) and information on exposures and potential confounders	p. 9 + Suppl. Table S1	Table 5 reports cohort size, age range, sex, C3GN/DDD subtype, treatment dose, sampling interval, and acquired/genetic disease drivers.
		(b) Indicate number of participants with missing data for each variable of interest	pp. 4-5	No biomarker values were missing. Tables 2–3 state a paired sample size of n=9 for each analyte in each cohort.

		(c) *Cohort study*—Summarise follow-up time (eg, average and total amount)	p. 2	Post-treatment sampling occurred approximately 3 months after treatment initiation in both cohorts.
Outcome data	15*	*Cohort study*—Report numbers of outcome events or summary measures over time	pp. 4-5, 9-10 + Suppl Table S1	Tables 2–3 report cohort-level paired biomarker changes; Table 4 reports patient-level PD classifications; Table 6 reports the descriptive Peg#6 treatment-switch profile; Supplementary Table S1 reports raw paired patient-level values.
		Case-control study—Report numbers in each exposure category, or summary measures of exposure	N/A	
		Cross-sectional study—Report numbers of outcome events or summary measures	N/A	
Main results	16	(*a*) Give unadjusted estimates and, if applicable, confounder adjusted estimates and their precision (*eg*, 95% confidence interval). Make clear which confounders were adjusted for and why they were included	pp. 4-5	Unadjusted pretreatment/posttreatment values, percent changes, and P/q values are reported in Tables 2–3; no confounder-adjusted models were performed.
		(*b*) Report category boundaries when continuous variables were categorized	pp. 4-5	The actual PD thresholds are on p. 4: iptacopan C3 ≥90 mg/dl and exploratory pegcetacoplan C3 ≥250 mg/dl; assay-boundary handling is on p. 5.
		(*c*) If relevant, consider translating estimates of relative risk into absolute risk for a meaningful time period	N/A	
Other analyses	17	Report other analyses done—*eg* analyses of subgroups and interactions, and sensitivity analyses	pp. 5-8 and p.10	Other analyses included pegcetacoplan spiking and CP40 competition experiments, patient-level PDC/LPDC/PDD classification, and a descriptive post-transplant treatment-switch analysis in Peg#6. No formal subgroup, interaction, or sensitivity analyses were performed.
**Discussion**				
Key results	18	Summarise key results with reference to study objectives	pp. 9-10	The Discussion summarizes the shared suppression of AP and terminal pathway activity, the distinct mechanism-specific biomarker signatures, and the patient-level concordant and discordant profiles in relation to the study objectives.
Limitations	19	Discuss limitations of the study, taking into account sources of potential bias or imprecision. Discuss both direction and magnitude of any potential bias	p. 10	Limitations include the small nonrandomized cohort, uncontrolled treatment assignment, selection of participants with paired samples, unavailable clinical outcomes/adherence/pharmacokinetic and dose-timing data, use of assay-boundary values, development and application of the classification in the same cohort, and limited representativeness.

Interpretation	20	Give a cautious overall interpretation of results considering objectives, limitations, multiplicity of analyses, results from similar studies, and other relevant evidence	p. 10	Interpretation is explicitly pharmacodynamic rather than therapeutic. The manuscript avoids claims of comparative efficacy or treatment selection, considers existing mechanism-specific evidence, and states that clinical correlation and independent validation are required.
Generalisability	21	Discuss the generalisability (external validity) of the study results	p. 10	The manuscript states that findings from a specialized cohort and laboratory may not generalize across C3G populations, sampling schedules, treatment durations, or assay platforms and calls for independent prospective validation with standardized sampling.
**Other information**				
Funding	22	Give the source of funding and the role of the funders for the present study and, if applicable, for the original study on which the present article is based	p. 11	The study was partly supported by NIH grant R01 DK110023. The funding agency had no role in data analysis or the decision to submit the manuscript.

*Give information separately for cases and controls in case-control studies and, if applicable, for exposed and unexposed groups in cohort and cross-sectional studies.

Note: An Explanation and Elaboration article discusses each checklist item and gives methodological background and published examples of transparent reporting. The STROBE checklist is best used in conjunction with this article (freely available on the Web sites of PLoS Medicine at http://www.plosmedicine.org/, Annals of Internal Medicine at http://www.annals.org/, and Epidemiology at http://www.epidem.com/). Information on the STROBE Initiative is available at www.strobe-statement.org.
